# Development and Content Validation of a Nursing Clinical Simulation Scenario on Transfusion Reaction Management

**DOI:** 10.3390/ijerph21081042

**Published:** 2024-08-08

**Authors:** Francisco Mayron Morais Soares, Samia Valéria Ozorio Dutra, Gleiciane Kélen Lima, Ana Beatriz Frota Lima Rodrigues, Davi Santos Magalhães, Elaine Cristina Negri, Igor Cordeiro Mendes, Luciana Mara Monti Fonseca, Lucas Ribeiro Araujo, Maria Ivaneide Teixeira dos Santos, Ana Clara Negri, Ana Valeska Siebra e Silva, Tatyane Oliveira Rebouças, Carmen Heidi Linhares, Francisco Arnoldo Nunes de Miranda

**Affiliations:** 1 Department of Nursing, Federal University of Maranhão, Imperatriz 65915-240, MA, Brazil; lucas.ra@discente.ufma.br; 2 Nancy Atmospera-Walch School of Nursing, University of Hawaii at Manoa, Honolulu, HI 96822, USA; samiaval@hawaii.edu (S.V.O.D.); carmen.linhares@hawaii.edu (C.H.L.); 3 Department of Nursing, State University of Ceará, Fortaleza 60714-903, CE, Brazil; gleicianeklima@gmail.com (G.K.L.); igor.mendes@uece.br (I.C.M.); ana.valeska@uece.br (A.V.S.e.S.); tatyane.oliveira@hemoce.ce.gov.br (T.O.R.); 4 Department of Nursing, INTA Uninta University Center, Itapipoca 62600-000, CE, Brazil; enferbia17@gmail.com (A.B.F.L.R.); davisantos1121246@gmail.com (D.S.M.); ivaneides2018@gmail.com (M.I.T.d.S.); 5 Department of Nursing, University of West Paulista, Presidente Prudente 19050-920, SP, Brazil; elainenegrisantos@gmail.com (E.C.N.); anacnegri@icloud.com (A.C.N.); 6 Escola de Enfermagem de Ribeirão Preto, University of São Paulo, Ribeirão Preto 14040-902, SP, Brazil; lumonti@eerp.usp.br; 7 Department of Nursing, Federal University of Rio Grande do Norte, Natal 59078-970, RN, Brazil; francisco.miranda@ufrn.br

**Keywords:** transfusion reactions, nursing education, simulation

## Abstract

Blood transfusion is a life-saving procedure widely used in healthcare. However, complications such as transfusion reactions may occur. Knowledge of these reactions is essential for patient safety. Nurses play a crucial role in this process by identifying complications and adverse reactions early on. A lack of professional competence in blood transfusion can lead to errors and serious complications, such as death. The aim of this study was to present evidence of the content validity of a simulated clinical scenario on transfusion reactions for teaching and learning for nursing students. This methodological study was carried out in three phases: (1) development of the simulated scenario of a transfusion reaction; (2) analysis of evidence of content validity by experts (*n* = 11); and (3) determination of satisfaction and self-confidence in the use of the simulated scenario by the nursing students (*n* = 45). The Content Validity Index was 94%. After the scenario had been developed, the content was validated and approved by 100% of the experts. All the items in the simulated scenario obtained agreement scores above 0.90. The simulated scenario was validated in terms of content and can be used to teach the management of transfusion reactions.

## 1. Introduction

Blood transfusion is a common treatment used in the clinical environment [1]. It is an accepted approach worldwide for a variety of clinical treatments, such as surgeries and emergencies [2,3]. With each procedure carried out, the patient is subject to the risk of suffering adverse events, such as transfusion reactions [2].

Transfusion reactions are classified according to their severity. They are categorized as immediate if manifesting in the first 24 h following the start of the transfusion or late if manifesting after 24 h from the start of the transfusion. Any transfusion reaction in Brazil must be reported through the National Hemovigilance System to the National Health Surveillance Agency (ANVISA). According to the ANVISA, 84.21% of the reported transfusion reactions are immediate and mild [4]. 

Complications of transfusion reactions include fever hemolytic reactions, infection, transfusion-related acute lung injuries and graft–host disease [5]. Although transfusion reactions are inevitable, a large number of incidents relating to the act of transfusion result from human error [5,6]. Incorrect identification of the recipient and incorrect labeling of the sample are the most common errors, accounting for 80% of reported events. A total of 45% of deaths could be avoided [7,8] with the prevention of human errors.

In this context, considering that transfusion reactions present serious risks and reactions such as acute hemolysis, which can be fatal, professional nurses should be thoroughly educated in the transfusion process during their undergraduate studies to ensure safe patient care [1].

Measures to reduce the chances of human error may improve transfusion safety. Educational strategies, such as clinical simulations, can be an effective tool to achieve this goal. The Global Action Plan for Patient Safety 2021–2030 includes the use of strategies to increase the safety of healthcare [9,10].

Active teaching and learning methods in health training are widely discussed as they contribute to professional and personal development in order to make the learner an active agent of knowledge. One of these methods is clinical simulation, which is a teaching strategy combined with technology for the controlled reproduction of health situations. It is a pragmatic replication of an event in a controlled environment for the development of cognitive, psychomotor and behavioral skills, with multiple purposes [11]. 

The use of a clinical simulation is in line with the National Curriculum Guidelines for undergraduate nursing, encouraging its regular use in teaching. Replicating real health situations reflects social and pedagogical transformations, improves students’ professional skills, critical thinking, problem solving, team management and self-confidence and increases the value of their education [12].

Nursing students should be trained to develop the skills needed to care for patients in cases of transfusion reactions in preparation for the responsibility as future professionals managing blood transfusions. Valid clinical scenarios provide valuable resources, ensuring safety and control in teaching and learning and allowing objective and effective learning [13].

The *International Nursing Association for Clinical Simulation and Learning (INACSL)* emphasizes the use of rotations and theoretical references in the creation of scenarios, since simulations are close to reality, allowing evaluation and self-assessment based on the desired results [14]. 

In Brazil, there are similar simulated scenarios for developing nursing care competencies in various topics, including in the pediatric context for therapeutic play [15] and neonatal umbilical cord care [16] and in adult care for people with non-suicidal self-injuries [17] and women attending reproductive planning nursing consultations [18]. However, to date, there are no studies on simulation scenarios on the topic of blood transfusion, making this research study the first to adopt one. Therefore, this study focused on addressing this gap by developing and validating a transfusion reaction simulation.

In addition, clinical simulations of a transfusion reaction could be implemented for continuing education in the professional arena and could potentially reduce errors in the transfusion process. This study therefore set out to present evidence of the validity of the content of a simulated clinical scenario in transfusion reactions for the teaching and learning of nursing students.

## 2. Materials and Methods

### Study Design

This is a methodological study which developed and validated the content [19] of a simulated scenario for transfusion reaction management. The purpose of this type of methodological study is to develop, validate and evaluate research instruments and techniques, with the aim of developing a reliable instrument that can later be used by other researchers. To this end, this methodological study focuses on the development, evaluation and improvement of methodological instruments and strategies. To write the article, the *Revised Standards for Quality Improvement Reporting Excellence* (SQUIRE 2.0) guidelines were adopted [20]. This study was approved by the Research Ethics Committee of the Higher Education Institution to which it was linked, under case number 5.935.534/2023. All participants signed an informed consent form (ICF).

The simulation scenario for the management of transfusion reactions was developed in three distinct phases: (1) development of the simulated scenario on transfusion reactions; (2) analysis of evidence of content validity by experts; and (3) identification of satisfaction and self-confidence in the use of the simulated scenario by nursing students. The aim of phase 1 is to develop a clinical simulation scenario for the management of transfusion reactions. Phase 2 presents evidence of the validity and the level of agreement between experts. Finally, phase 3 aims to implement the scenario built and validated in the previous phases to verify the satisfaction and self-confidence of nursing students in learning. Below is more detailed information on the procedures adopted in each phase.

**Phase 1: Preparation of a simulated transfusion reaction scenario**

The process consisted of two stages: first, a protocol was used to search for and select the content used in the development of scenarios by means of a literature review of the PUBMED/MEDLINE, SCOPUS, LILACS and BDENF databases, using the following keywords: clinical simulation; teaching materials; hemotherapy; transfusion reaction; nursing; and hemotransfusion. 

We included primary studies whose authors presented information on transfusion reactions and their clinical management published in Portuguese, English or Spanish in the period between January 2020 and March 2023. While we excluded information from books and/or chapters, study descriptions of integrative reviews and systematic literature reviews and/or meta-analysis, as well as reflection articles. In addition to scientific articles, we consulted manuals from the government body that regulates and standardizes hemovigilance in Brazil, the National Health Surveillance Agency—ANVISA [4]. The time frame was established to guarantee up-to-date evidence of the literature on the subject.

In the subsequent stage, a list of terms, concepts and definitions was developed for the textual structuring of the scenario, respecting the items based on the recommendations proposed for the construction of scenarios [21]. These are (a) the student’s prior knowledge; (b) the learning objectives; (c) the activity’s theoretical basis; (d) preparing the scenario; (e) developing the scenario; (f) *debriefing*; and (g) evaluation. After preparing the scenario entitled Clinical Nursing Management of Transfusion Reactions, we proceeded to phase two to validate the content of the developed scenario.

**Phase 2: Analysis of content validity evidence by *experts***

At this stage, the researchers from the Research Group on Advanced Practices and Technologies in Nursing indicated the names and contacts of professionals eligible for the study from all regions of the country and, for selecting experts, considered the criteria used in a study [22] on the reflection in teaching, care and research involving the following areas of obstetric nursing and the emergence and validation of technologies, based on the database of the Directory of the National Council for Scientific and Technological Development (CNPq). They also used snowball sampling to indicate other professionals. This choice of participants was made by convenience. Thus, 31 professionals received invitations via email and instant messaging groups, and 11 of them responded, making up the study sample [23]. 

After agreeing to take part in the study, the expert evaluators were sent a link to the following documents:

− Simulated scenario built in phase 1, sent in a *Survey* format;
− Health Education Content Validation Instrument [24]: an instrument validated in the literature that contains 18 items organized into three domains: objectives, structure/presentation and relevance with scores of 0, disagree; 1, partially agree; and 2, totally agree;
− The informed consent form (ICF).

The Content Validity Index (CVI) was applied to verify evidence of the content validity of the simulated scenario, based on the scores of the health education content validation instrument assigned by the experts. The Content Validity Index measures the proportion or percentage of experts whose opinions agree on certain aspects of the instrument and its items. An index above 0.80 was considered valid [25]. The index score for each item was calculated using the sum of the items marked as 2 (totally agree) by the experts, divided by the total number of responses. 

The proportion of agreement greater than or equal to 0.80 was judged to determine the evidence of validity. In order to verify whether the proportion of agreement between the experts was statistically equal to or greater than 0.80, the binominal test was applied, and the significance level adopted was *p* > 0.05, corroborating other studies [11,12,13,14,15,16,17,18,19,20,21,22,23,24,25].

**Phase 3: Identification of satisfaction and self-confidence in the use of the simulated scenario by nursing students**

The aim of this stage was to assess the acceptability of the simulation using the satisfaction and self-confidence instrument. The instrument was developed by the National League for Nursing (NLN) and translated and validated into Portuguese. The instrument contains 5 satisfaction items and 8 self-confidence items, with a total of 13 items. Responses are recorded on a Likert scale ranging from 1 (strongly disagree) to 5 (strongly agree). The total score ranges from 13 to 65 points. The instruments showed a high degree of internal consistency in this study (Cronbach’s alpha 0.84) [26]. 

After validating the content of the scenario, a pilot implementation was carried out with five students who did not take part in the study. The convenience sample was derived from fifth-year students enrolled in a bachelor’s degree in nursing, aged 18 or over and with no previous experience of managing a transfusion reaction, who were invited to take part in the scenario. McNemar’s chi-square test formula was used to check whether the sample was adequate for the power of the study. The test showed satisfactory results, with values of 91.4% for the power of study, following recommendations in the literature that suggest a cut-off point for a power of study of over 80% [27]. We also calculated the Intraclass Correlation Coefficient (ICC), which showed excellent results according to the literature [25].

Groups of five students took part in the transfusion reaction simulation clinical scenario, which was implemented nine times (*n* = 45). They were also able to experience the scenario with the support of evaluation checklists, which they filled in during development. The researcher moderated the stages using the theoretical framework [21] to conduct the scenario and its stages [17]. The transfusion reaction simulation scenario lasted 15 min, after which the students took part in a structured debriefing lasting around 30 min.

## 3. Results

### 3.1. Drawing Up the Scenario

The clinical simulation scenario on transfusion reactions was designed according to the items mentioned in Table 1 and was guided by the learning objectives, respecting the items based on the recommendations proposed for the construction of scenarios [21].

### 3.2. Evidence of the Content Validity of the Simulated Scenario

The 11 experts were from five regions of Brazil and had more than 10 years of experience in blood transfusion. One (9%) had a master’s degree, and ten (91.0%) had a doctorate in nursing and related areas; of these, nine were faculty staff. The group of experts analyzed evidence of the content validity of the simulated scenario

According to Table 2, the agreement between the experts was satisfactory (*p*-value > 0.05), reaffirming the content validation, structure, presentation, and relevance. There was therefore no need to further change the scenario content to meet the proposed objectives. 

No item had a Content Validity Index below the cut-off score. This shows the high internal consistency of the assessment and reinforces the validation of the content of the simulated scenario script. The only items with a slight disagreement were 3 and 5; in their justification, the expert said that the item could only be evaluated if there was an intervention study measuring behavioral changes. The clinical simulation scenario obtained a total content validation index of 0.94, indicating that the content was validated by the nursing experts. 

### 3.3. Identification of Satisfaction and Self-Confidence in the Use of the Simulated Scenario by Nursing Students

Forty-five students completed the instrument to assess satisfaction and self-confidence in learning. They were nursing students in a baccalaureate program enrolled in the fifth semester, predominantly females (80%), with an average age of 22.6 years old. The evaluation of satisfaction and self-confidence is described in Table 3.

All the items in Table 3 on satisfaction and self-confidence showed significant results of over 60% agreement. These data show significant and high levels of satisfaction and confidence in the use of simulation as a teaching and learning method. The statistical analysis of *Cronbach*’s alpha showed a high internal consistency. The scale’s internal consistency was highly reliable (Cronbach’s α = 0.95 for satisfaction and 0.91 for self-confidence), and the Intraclass Correlation Coefficient (ICC) scores are shown in Table 4, which show an excellent level of agreement between the observers [25].

## 4. Discussion

The simulation scenario that was developed is relevant to the initial care of patients with transfusion reactions in an acute care unit, considering that clinical management must be carried out by qualified teams to allow initial propaedeutic and therapeutic interventions to begin [2]. Our simulated scenario improved the existing scientific body of knowledge addressing theoretical and practical issues [7,8]. Other studies assessed knowledge improvement through scenarios focused on the continuous professional training of nurses [31], active searches for health agents for patients with leprosy [32], and the practice of anesthesia [33]. Studies involving the development of simulated scenarios on transfusion reactions are scarce in the literature. Ours, as the first study on the subject, showed content validity with indices above 0.80, proving the simulation to be suitable for use in teaching and learning.

A good-quality simulated scenario needs to be developed based on the scientific evidence available in the literature and then validated by experts who are proficient in the subject. In our study, the expert nurses who performed the content validation had more than ten years of professional experience in blood transfusion and health technology, as well as being faculty staff, which corroborated other studies’ validation processes [34,35].

The content validation process has also been used in other scenarios such as newborn umbilical cord care [16], cardiopulmonary resuscitation [13], sepsis management [36] and nurse care management in a hospital setting [37]. Our scenario had a high Content Validity Index (CVI) (CVI per item above 0.80) and was conducive to achieving the learning objectives.

Additionally, we verified the satisfaction and self-confidence in the learning of the nursing students and obtained an excellent index of agreement among the students (Cronbach’s α = 0.95 for satisfaction and 0.91 for self-confidence in learning and Intraclass Correlation Coefficients of 0.85 and 0.80). These results corroborate the increase in satisfaction and self-confidence in simulation-mediated learning [38], pediatric nursing [39], clinical management of difficult conversations in oncology [40], oncological emergencies [41], clinical management of individuals with COVID-19 [42], and management of blood transfusion in babies with hyperbilirubinemia [43].

The limitations of the study include a scarcity number of studies on the clinical management of transfusion reactions, the difficulty in recruiting experts on the subject, the timely validation of the content of the studies, and the convenience sampling for the implementation of the scenario. Future studies should consider the process of determining validity and reliability, as there are various methods to develop valid and reliable simulated scenarios [44], keeping in mind the definition of validation for simulations [45].

## 5. Conclusions

The clinical simulation scenario for managing transfusion reactions was validated with regard to content and provides a proper training strategy for students to manage transfusion reactions appropriately, using the principles of patient safety. Additionally, it obtained an excellent rate of satisfaction and self-confidence in learning.

This study contributes to the field of nursing and health by offering the academic and professional community a validated scenario on transfusion reactions, achieving quality teaching for safe care. We also suggest future research to verify the clinical effectiveness of the scenario for increasing the knowledge of nursing students and professionals.

## Figures and Tables

**Table 1:** Clinical simulation scenario for transfusion reactions.

Scenario identification		
Theme	Transfusion Reaction.	
**Learning objectives**		
**Upon completion of this activity, the participant should be able to:** Supervise the patient’s blood transfusion process. Prescribe blood transfusion for the patient. Assess vital signs before and after administering the blood bag. Identify the signs and symptoms of a transfusion reaction. Acquire technical skills in the management of patients with transfusion reactions.		
**Material resources**		
Environmental	Occurrence of Transfusion Reactions in a Clinic	
Simulators	Medium fidelity simulator	
Human Resources 2 actors 5 participants	Actor 1: the victim’s relative, who will ask for help. Actor 2: the patient exhibiting a reaction. Participant 1: a blood bank nurse. Participant 2: a blood bank doctor. Participant 3: doctor 1 at the clinic. Participant 4: doctor 2 at the clinic. Participant 5: vital signs function. Participant 6: blood bag installation nurse.	
**Materials used**	**Monitoring material**	
	Venturi kit (face mask, corrugated trachea, extension for connection to the flow meter, humidification/inhalation adapter, six colored valves for different FiO_2_ concentrations—orange, pink, green, white, yellow and blue). Sterile distilled water. Gas ruler (oxygen, compressed air and vacuum). Humidifier with water.	
	**Cardiopulmonary Resuscitation Material**	
	One simulation mannequin—torso; One electrocardiogram monitor; Monitoring cable; Electrodes; Conductive gel; Sensor for oximetry; Intermediate cable; Monitor or display; Personal protective equipment; Oxygen 100% at 15 L/min; Cardiopulmonary Resuscitation Cardiorespiratory Arrest Trolley, precisely equipped with: Manual respirator with a reservoir; Orotracheal tube (No. 7.0, 7.5, 8.0 or 8.5); Intubation material (laryngoscope handle and blade no. 2.0, 3.0, 4.0, 5.0 and guide); Suction equipment (aspirator and tracheal tube (suction) no. 12 or 14); Cardiac massage board; Defibrillator/cardiac monitor and electrodes; Conductive gel; Medication (adrenaline, amiodarone, fentanyl, saline 0.9%); 20 mL, 10 mL and 05 mL syringes; IV line; Needles (40 × 12 and 30 × 8); Sterile glove No. 7.5, 8.0 or 8.5; Procedure gloves; Shoelace to secure the tube; Scalp no. 19, intravenous device (jelco) no. 14, 16 or 18.	
	**Transfusion material**	
	Personal protective equipment (gloves, cap, surgical mask and lab coat); Thermometer; Stethoscope; Sphygmomanometer; Tray; Cotton/gauze; 70% alcohol; Tourniquet; Peripheral intravenous catheter (jelco); Bandage; Transfusion equipment; IV stand; Blood component bag (red blood cell concentrate, fresh plasma, cryoprecipitate and/or platelets) with identification label; Transfusion evolution sheets; Physiological saline (SF) at 0.9% (500 mL).	
**Scenario description for the Instructor**		
Presentation	Actor pretending to be the victim’s relative: “Doctor, *he’s my son. I spoke to him, but he wasn’t answering me, just moaning about being in pain and shivering. Please help me*”. Physical examination (if assessed): Patient presents with hyperthermia and tachycardia.	
Operator-defined parameters	HR: 75 bpm (after installation: 110 bpm); FR: 18 rpm (after installation: 22 rpm); BP: 120 × 80 mmHg (after installation: 160 × 100 mmHg); T: 36.5° (after installation: 38.9°).	
Operator interventions	Action	Reaction
	Participant observes chills and tachycardia	Call for help
	Patient checks vital signs	Altered vital signs Stop the drip
	Patient begins to feel ill	Companion: “Screams” Taken out of the emergency room by the technician
	Summoning the installation team	Administer through SF 0.9% venous access Request service from the hematology team
	Conducting transfusion reaction care	End of scene
Case characterization		
**Secondary Assessment with the following information:** 21-year-old male patient, A positive. Allergy-free Taking iron and vitamin B12 Patient has profound anemia My last meal was in the morning: a glass of juice and a piece of cake.		
**Scenario context**		
Pre-briefing	**For everyone involved:** we need five people for the service.	
	**For participants only:** The nursing team has been called to perform a blood transfusion on a 21-year-old male patient admitted to clinic 2 in bed 17. When they come across the scene, they need to check the transfusion parameters (temperature, heart rate, respiratory rate and blood pressure). Then, they need to carry out the correct administration process, and, if there are any changes, the blood bank nurse and the doctor on duty should be informed. You have all the necessary materials for use.	
	**For observers only:** They will watch the service as spectators. At the end, the facilitator will ask the two observers to *debrief on the* learning they observed.	
**Theoretical foundation**		
Protocol from the National Health Surveillance Agency [4]		
**Bibliographical references**		
Heroes, A.-S.; Kabamba, P.; Luyindula, A.; Bongenya, B.; Nzazi, P.; Nasali, M.; Akele, C.; Lusinga, M.-P.; Ekofo, J.; Coene, J.; et al. Knowledge, Attitude and Practice Survey of Bacterial Contamination of Blood for Transfusion in the Democratic Republic of the Congo. *Blood Transfus* 2023, doi:10.2450/BloodTransfus.586. [3] Lea, N.C.; Gibbs, K.; Johnson, C.; Lam, A.; Wuestner, E.; Hui, S.-K.R. Transfusion-Associated Adverse Events. *Journal of Infusion Nursing* 2022, 45, 264–269, doi:10.1097/NAN.0000000000000483. [28] Miao, W.; Sibbald, S.L.; Law, B.; Solh, Z. Understanding the Nursing Practices and Perspectives of Transfusion Reaction Reporting. *J Clin Nurs* 2023, 32, 1045–1052, doi:10.1111/jocn.16310. [2] Ministry of Health (BR) National Helth Surveillance Agency. Hemovigilance Report 2015. 2015. [4] Silveira, A.C.D.A. da; Santos, L.M.S. dos; Costa, P.C.F.; Braga, M.D.N. da S.; Borges, T.D.S.; Costa, A.G. da Sistematização Da Assistência de Enfermagem Segundo o Conhecimento de Enfermeiros do Ambulatório de Um Hemocentro. Revista de Enfermagem da UFSM 2021, 11, e69, doi:10.5902/2179769264111. [29] Suddock, J.T.; Crookston, K.P. *Transfusion Reactions*; 2024. [30]		
**Remarks for the instructor**		
The scenario should unfold within 15 min. The instructor should call in the nursing team if required. The following questions must be answered: ▪ Did they perform proper hand hygiene? ▪ Did they manage the patient with a transfusion reaction properly? ▪ Did they stop the drip immediately? ▪ Did they call the doctor? ▪ Did they call the nurse? ▪ Did they consult the hematology team? ▪ Did they demonstrate teamwork, leadership and communication?		
**End of scene**		

**Table 2:** Evidence of the content validity of the simulated scenario.

Items Evaluated	Content Validity Index per Item (CVI-I)	*p* *
**Objectives**		
1. Contemplates proposed theme	0.97	0.98
2. Suitable for the teaching–learning process	0.95	0.97
3. Clears up doubts about the subject matter	0.89	0.91
4. Enables reflection on the topic	1.00	1
5. Encourages behavioral changes	0.89	0.91
**Structure and Presentation**		
6. Language appropriate to the target audience	1.00	1
7. Language appropriate to the educational material	0.95	0.97
8. Interactive language, allowing active involvement in the educational process	0.92	0.89
9. Correct information	0.97	0.98
10. Objective information	0.95	0.97
11. Clarifying information	0.95	0.97
12. Necessary information	0.92	0.89
13. Logical sequence of ideas	0.95	0.97
14. Current topic	1.00	1
15. Appropriate text size	0.95	0.97
**Relevance**		
16. Stimulates learning	1.00	1
17. Contributes to knowledge in the field	0.97	0.98
18. Sparks interest in the topic	0.97	0.98
**CVI-T**	0.94	

* *p* > 0.05 Concordance binomial test; Content Validity Index for each item (CVI-I) and total CVI (CVI-T).

**Table 3:** Evaluation of the satisfaction and self-confidence of fifth-semester students (*n* = 45).

Item	Strong Disagree	Disagree	Neither Agree or Disagree	Agree	Strong Agree
	%				
Satisfaction *					
1. The teaching methods used in this simulation were useful and effective.				26.7	73.3
2. The simulation provided me with a variety of teaching materials and activities to promote my learning of the medical–surgical curriculum.				80.0	13.3
3. I liked the way my teacher taught through the simulation.				70.0	30.0
4. The teaching materials used in this simulation were motivating and helped me learn.				26.7	73.3
5. The way my teacher taught through the simulation was suitable for the way I learn.				26.7	73.3
Self-confidence **					
6. I am confident that I have mastered the content of the simulation activity that my teacher has given me.			6.7	66.7	26.7
7. I am confident that this simulation has included the necessary content for mastering the medical–surgical curriculum.				90.0	10.0
8. I am confident that I am developing the skills and gaining the knowledge required from this simulation to perform the necessary procedures in a clinical environment.				80.0	20.0
9. My teacher used useful resources to teach the simulation.				30.0	70.0
10. It’s my responsibility as the student to learn what I need to know through the simulation activity.				60.0	40.0
11. I know how to get help when I don’t understand the concepts covered in the simulation.				26.7	76.3
12. I know how to use simulation activities to learn skills.				40.0	60.0
13. It is the teacher’s responsibility to tell me what I need to learn about the topic developed in the simulation during the lesson.				30.0	70.0

* Cronbach’s alpha, satisfaction: 0.95; ** Cronbach’s alpha, self-confidence in learning: 0.91.

**Table 4:** Reliability of evaluations of satisfaction and self-confidence of fifth-semester students (*n* = 45).

Variable	ICC	CI (95%)
Learning satisfaction	0.84	0.65–0.91
Self-confidence in learning	0.80	0.61–0.90

## Data Availability

The data supporting the findings of this study are available from the corresponding author upon request.
